# Comparison of radiological spino-pelvic sagittal parameters in skiers and non-athletes

**DOI:** 10.1186/s13018-015-0305-6

**Published:** 2015-10-17

**Authors:** Carl Todd, Peter Kovac, Anna Swärd, Cecilia Agnvall, Leif Swärd, Jon Karlsson, Adad Baranto

**Affiliations:** Department of Orthopaedics, University of Gothenburg and Sahlgrenska University Hospital, Gothenburg, Sweden; Department of Radiology, Institute of Clinical Sciences at Sahlgrenska Academy, University of Gothenburg and Sahlgrenska University Hospital, Gothenburg, Sweden; Sportsmedicine Åre and Åre Ski High School, Gothenburg, Sweden; The Carl Todd Clinic, 5 Pickwick Park, Park Lane, Corsham, SN13 0HN UK

**Keywords:** Athletes, Pelvic parameters, Radiological, Spinal curvatures, Spino-pelvic alignment

## Abstract

**Background:**

The purpose of the present study is to compare the radiological parameters of the spino-pelvic sagittal alignment in young elite skiers and non-athletes of a similar age.

**Methods:**

The sample group (*n = 102)* consisted of elite Alpine and Mogul skiers (*n = 75)* and a non-athletic population (*n* = 27), mean age for both groups was 17.7 (±1.4) years (skiers mean age 18.3 SD 1.1 and controls 16.4 SD 0.6). Radiological measurements of the spino-pelvic sagittal alignment were examined from plain radiographs taken in the long-standing position.

**Results:**

There were no significant differences reported in the pelvic parameters between both groups. A difference was reported in the sagittal vertebral axis between skiers (8.0 cm SD 46.0) and the control group (−2.0 cm SD 39.0), which may be of clinical significance, in spite of being statistically non-significant. Type I spinal curves according to Roussouly were shown to be more prevalent in the skiers (18.2 %) compared with the control group (0.0 %) and were statistically significant (*p* = 0.03).

**Conclusion:**

Elite young skiers are shown to have a more prevalent type I spine and a different spino-pelvic sagittal alignment compared to a healthy non-sporting population of a similar age.

## Background

Spino-pelvic sagittal alignment is maintained by the pelvic girdle facilitating the balance of lumbar lordosis with hip joint extension to regulate and maintain humans in an upright stable posture [1–8]. Previous studies have evaluated spino-pelvic sagittal alignment using plain radiographs [1, 5, 7, 9–21]. It has been proposed that radiographic evaluation of pelvic parameters, spinal curvatures and global balance [21] may help to characterise the morphology and functionality of the spine and pelvis [3, 22].

**Fig. 1:**
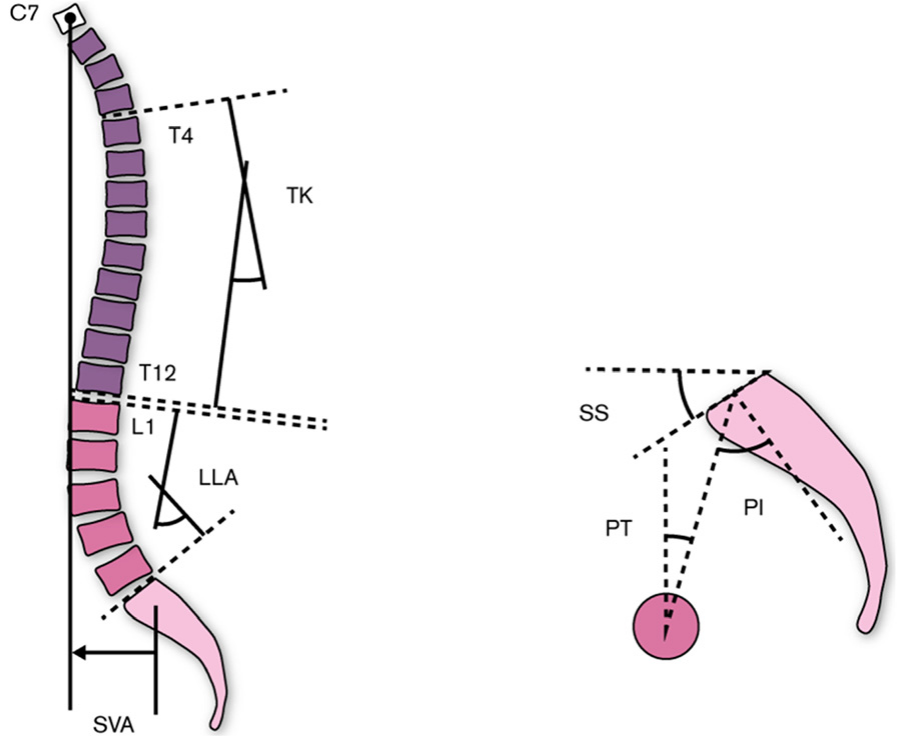
Showing relation between spinal curves, pelvic parameters and global balance (adapted from Roussouly et al. [26])

**Fig. 2:**
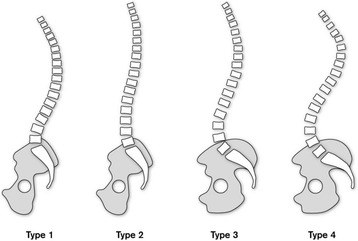
Spinal curvatures (adapted from Roussouly et al. [26])

The spine has several curvatures in the sagittal plane, a cranial and caudal lordotic curve that is separated by the kyphotic curve [4]. These curvatures develop from changes associated with growth, balance and posture [11] to divide the distribution of forces evenly throughout the spinal column [18–21]. The curvatures are intrinsically related and have been shown to influence the form and function of the pelvis and hips [1, 5, 9]. Spinal curvatures have also been categorised by morphological and positional measurements that help to determine the pelvic parameters (Fig. 1). Moreover, specific spinal pathologies have been attributed to three of the four types of spinal curvatures (Fig. 2) according to Roussouly and Pinheiro-Franco [5]. These range from increased disc degeneration in the thoracolumbar region with type I to central disc herniation in type II, a well-balanced spine with type III and an increased risk of spondylolisthesis in type IV [23–25]. A correlation has also been shown to exist between the pelvic parameters and the four types of spinal curvatures [26]. Moreover, an individual’s head position in relation to their normal centre of gravity provides an overview of global spinal balance [4, 27].

Previous studies have shown sporting participation to be associated with changes in the spino-pelvic sagittal alignment of athletes [28–30]. However, the quality of these studies is limited as sagittal alignment was evaluated with non-radiological methods. Poor levels of validity have been shown for correlation of non-radiological and radiological evaluation of spinal sagittal alignment [31, 32]. Therefore, further research for evaluation of the radiological spino-pelvic parameters between athletes and non-athletes appears reasonable.

The purpose of the present study is to compare the radiological spino-pelvic sagittal parameters between young elite skiers to those of a healthy non-athletic population of a similar age. The hypothesis of the present study is to show that the spino-pelvic sagittal alignment of young elite skiers is different to that of a healthy non-athletic population. To our knowledge, this is the first study of this type to carry out such an investigation.

## Methods

The sample group (*n = 102)* consisted of young athletic elite Alpine and Mogul skiers (*n = 75)* and a non-athletic population (*n* = 27). All pupils (grades 1–4, between 16 and 20 years of age) attending the Åre High School Ski Academy, Sweden, were invited to participate in this prospective study after a short presentation about the project by two of the authors. The testing was carried out at the same school, and the radiographic examinations were taken at the Radiographic Department, Östersund Hospital, Sweden.

Inclusion criteria for the control group was first year high school pupils from a class at a High School in Östersund, Sweden, that have not previously or at present participated in any organised sporting activities for more than 2 h per week. Participants (skiers and controls) were excluded if they had an episode of low back, pelvic or hip pain and history of previous surgery to the lumbar spine, pelvis or hip joint or a history of systemic pathology including inflammatory arthritis or pelvic inflammatory disorders or if they were pregnant. The demographic characteristics of the full sample are presented in Table 1.

**Table 1:** Baseline characteristics for all subjects and stratified by group

	All subjects (*n* = 102)	Skiers (*n* = 75)	Controls (*n* = 27)	*p* value
Age (years)	17.7 (1.4)	18.3 (1.1)	16.4 (0.6)	<0.001
Female sex, *n* (%)	53 (52 %)	35 (47 %)	18 (67 %)	0.074
Height (cm)	173 (8.3)	174 (8.2)	172 (8.6)	0.19
Weight (kg)	69 (12.2)	70 (9.1)	67 (17.9)	0.39
Body mass index (kg/m^2^)	22.9 (3.3)	22.9 (2.2)	22.7 (5.3)	0.81

Values are mean and standard deviation (SD)

The present study was approved by the Regional Ethical Review Board in Gothenburg at The Sahlgrenska Academy, Gothenburg University, Gothenburg, Sweden (ID number: 692-13).

### Testing procedure

#### Plain radiographic examination

For radiographic examinations, a standardised protocol was used for all participants [7]. Frontal and lateral long-standing plain radiographs recorded from C7 to the femoral head were obtained for each participant. Participants were instructed to stand with feet together in a natural upright posture, without spinal rotation, with arms hanging by their side for frontal views and arms horizontal resting on supports for sagittal views. The total measurement time was approximately 10 min. Automatic exposure control (AEC) was completed using a low dose, and the edges of the images were enhanced to clearly distinguish vertebral bodies and endplates. Radiographic images were taken from the C7 vertebrae to the femoral head; these were overlapped and automatically stitched for ease of interpretation. To reduce radiation levels, the film focus distance (FFD) was increased to 120 cm [33]. Frontal view with posterior-anterior (PA) beam direction, the entire vertebral bodies and half the femoral head were imaged. Lateral view with the beam direction from right to left, the entire vertebral bodies and half the femoral head were imaged. The entire vertebral bodies and the entire femoral head were imaged. The radiographs were measured for sagittal spinal curvatures by a single blinded experienced radiologist with the angular parameters reported in degrees. A negative value (−) represented a lordotic alignment whilst a positive value (+) represented a kyphotic alignment. Geometrical measurements relating to spinal curvatures were obtained from the following: thoracic kyphosis (TK) (Fig. 1) was defined as the angle measured from the upper endplate of T4 to the lower endplate of T12, and lumbar lordosis (LL) (Fig. 1) was defined as the angle measured from the upper endplate of L1 to the upper endplate of S1. Previous studies have shown good reliability for radiographic evaluation of spinal curvatures [34, 35].

#### Pelvic parameters

Geometrical measurements relating to the pelvic parameters (Fig. 1) were measured and recorded in degrees from the following. Pelvic incidence (PI) is a morphological parameter and is the angle measured from a perpendicular line to the mid-point of the sacral plate and extended to the centre of the femoral head. Pelvic tilt (PT) is a positional parameter and is the angle measured from a perpendicular line starting at the centre of the femoral head and extended to the mid-point of the sacral plate. Sacral slope (SS) is a positional parameter and is the angle measured from the superior endplate of S1 and a horizontal axis [10, 26]. A geometrical relationship exists between the morphological (PI) and functional parameters (PT, SS) resulting in the equation PI = PT + SS [26].

#### Sagittal vertical axis

The sagittal vertical axis (SVA) (Fig. 1) was measured and recorded in centimeter and is defined by using the C7 plumb line that intersects the superior corner of the upper sacral endplate. The sagittal vertical axis assesses if an individual is in neutral, positive or negative alignment by comparing the head position relative to the sacral promontory [27].

#### Spinal curvatures

Four types of spinal curvatures correlating to the angle of the sacral slope were defined according to Roussouly et al. [26]. Type I: low sacral slope <35° with an 80:20 thoracolumbar curve. Type II: low sacral slope <35° with a 60:40 thoracolumbar flat back. Type III: sacral slope >35° <45° with a 50:50 thoracolumbar curve. Type IV: high sacral slope >45° with a 20:80 reversed thoracolumbar curve (Fig. 2).

### Statistical analysis

Data was analysed using IBM SPSS Statistics for Windows, Version 22.0. Armonk, NY: IBM Corp. The description of data was expressed in terms of the mean and standard deviation (SD), median and range, including frequencies and percentages as appropriate. An independent *t* test was performed to compare variables (skiers and controls). Fisher’s exact test was performed to compare the distribution of spinal curves according to Roussouly et al. [26] between variables. The statistical significance for all tests was set as *p* < 0.05.

## Results

Due to drop-out and failure to attend investigations, radiological data from 92 *(n =* 102) participants was only available for final analysis. Reasons given were difficulties with timings for radiology appointments, athletes travelling abroad and participant’s being worried about claustrophobia. Table 1 summarises the demographic characteristics of the whole population. The mean age of enrolled population was 17.7 (±1.4) years (skiers mean age 18.3 SD 1.1 and controls 16.4 SD 0.6, *p =* 0.001). Table 2 shows the radiology frequencies for all participants. Values for comparison of radiology between skiers and controls are presented in Table 3. Similar values were shown for comparison between both groups; however, the skiers’ mean SVA was 8.0 cm (SD 46) and the control group was −2.0 cm (SD 39). No statistical significances were noted for comparison between both groups with an independent *t* test (PI *p* = 0.794, PT *p* = 0.139, SS *p* = 0.587, SVA *p* = 0.361, TK *p* = 0.197 and LL *p* = 0.283). Table 4 shows the distribution between genders for pelvic parameters and spinal curvatures. Moreover, there were no significant differences shown for comparison between genders (PI *p* = 0.192, PT *p* = 0.461, SS *p* = 0.088, SVA *p* = 0.155, TK *p* = 0.400 and LL *p* = 0.474).

**Table 2:** Frequencies of all subjects for radiology

		PI	PT	SS	SVA	Thoracic kyphosis° (radiological)	Lumbar lordosis° (radiological)
*n*	Valid	92	92	92	89	92	92
	Missing	10	10	10	13	10	10
Mean		50.7	10	41.9	5.2	35.8	−59.1
Median		50	10	41	5	37	−58
SD		11.4	8.6	7.7	44.3	7.3	9.8
Range		66	62	35	202	40	44
Minimum		19	−7	23	−97	12	−82
Maximum		85	55	58	105	52	−38

Values are mean, median and standard deviation (SD) unless specified otherwise

**Table 3:** Comparison between skiers and controls for pelvic parameters and spinal curvatures

	Group	*n*	Mean	SD	*p* value
PI	Skiers	66	50.9	12	0.794
	Control	26	50.2	9.8	
PT	Skiers	66	10.9	9.2	0.139
	Control	26	7.9	6.3	
SS	Skiers	66	41.2	9.1	0.587
	Control	26	42.3	8.1	
SVA	Skiers	64	8	46	0.361
	Control	25	−2	39	
TK	Skiers	66	35	7	0.197
	Control	26	37	7	
LL	Skiers	66	−58.4	9.3	0.283
	Control	26	−60.9	11	

Values are mean, median and standard deviation (SD) unless specified otherwise

*PI* pelvic incidence, *PT* pelvic tilt, *SS* sacral slope, *SVA* sagittal vertebral axis, *TK* thoracic kyphosis, *LL* lumbar lordosis

**Table 4:** Distribution of pelvic parameters and spinal curvatures by gender

	Gender	*n*	Mean	SD	*p* value
PI	Female	47	49.2	13.5	0.192
	Male	45	50.3	8.5	
PT	Female	47	10.7	10.6	0.461
	Male	45	9.4	5.7	
SS	Female	47	39.9	10.3	0.088
	Male	45	43	6.7	
SVA	Female	47	8	46	0.155
	Male	45	−1.6	35.6	
TK	Female	47	35.4	6.8	0.400
	Male	45	35.1	7.9	
LL	Female	47	−59.9	10.6	0.474
	Male	45	−58.4	9	

Values are mean, median and standard deviation (SD) unless specified otherwise

*PI* pelvic incidence, *PT* pelvic tilt, *SS* sacral slope, *SVA* sagittal vertebral axis, *TK* thoracic kyphosis, *LL* lumbar lordosis

Table 5 shows the distribution of spinal curves between skiers and controls according to Roussouly et al. [26]. A type I spinal curve was shown to be more prominent in the skiers, 18.2 % (*n* = 12), compared with the control group, 0.0 % (*n* = 0, *p* = 0.03). Similarly, type II spinal curves were shown to be more prominent with the control group, 15.4 % (*n* = 4), compared with the skiers, 4.5 % (*n* = 3); types III and IV spinal curves were evenly distributed between both groups. Table 6 shows the distribution between the genders for the spinal curves according to Roussouly et al. [26]. No significant significance was shown between the genders for each group (*p* = 0.316).

**Table 5:** Distribution of Roussouly type for skiers and controls

Roussouly type	Skiers	Controls	*p* value^a^
1	12 (18.2)	0 (0.0)	0.030
2	3 (4.5)	4 (15.4)	
3	39 (59.1)	18 (65.4)	
4	12 (18.2)	5 (19.2)	

Number with column percentage in parenthesis

^a^Fisher exact test (38 % of cells analysed have expected cell counts less than 5)

**Table 6:** Distribution between genders for spinal curve type according to Roussouly et al. [26]

Spinal type curve	Female (*n* and %)	Male (*n* and %)	Total (*n* and %)
I	6 (12.8 %)	6 (13.3 %)	12 (13 %)
II	4 (8.5 %)	3 (6.7 %)	7 (7.6 %)
III	25 (53.2 %)	31 (68.9 %)	56 (60.9 %)
IV	12 (25.5 %)	5 (11.1 %)	17 (18.5 %)

## Discussion

The most important finding in this study is the greater difference in the type I spinal curvatures according to Roussouly et al. [26] in young skiers compared with controls of a similar age. Moreover, the SVA of the skiers demonstrated a greater difference compared with the controls. Therefore, we conclude that these differences with the type I spinal curves and SVA occur more often in skiers and may be a result of progressive loading from functional sports specifics rather than any particular structural alignment issue.

In the present study, the mean values of all participants for radiology pertaining to pelvic parameters have shown the PI (50.7°), PT (10°), SS (41.9°) and SVA (5.2 cm) to be similar to those previously reported within a normal asymptomatic population [3, 10, 13, 15, 25–27, 34, 36–38].

The mean values for the radiological measurements of TK (35.8°) and LL (−59.1°) in the present study were also similar to those previously reported in a normal asymptomatic population [4, 5, 9, 11, 18–21, 39, 40]. There were no significant differences noted for radiological comparison of both groups, i.e. skiers versus controls. Radiological values were similar for the PI of the skiers (50.9°) and controls (50.2°), the PT of the skiers (10.9°) and controls (7.9°) and the SS of the skiers (41.7°) and controls (42.3°). A difference was noted with the radiological value for the SVA of the skiers (8.0 cm) compared with the controls (−2.0 cm).

This may be of clinical relevance, in spite of being statistically non-significant, and suggest that within a younger population, sports such as skiing that require a predominance of forward-bending postures may be associated with functional changes that affect the global spinal balance rather than a specific structural issue. Values for the TK of the skiers (35.2°) and controls (37.4°) and LL of the skiers (−58.4°) and controls (−60.9°) were similar. There were no significant differences between genders for the values of pelvic parameters and spinal curvatures, which is similar to previous studies [10, 41].

A significant difference was noted for comparison between groups for spinal curves according to Roussouly et al. [26]. Type I spinal curves were shown to be more prominent and statistically significant for the skiers (18.2 %) compared with the control group (0 %). Type II spinal curvatures were shown to be more common in the control group (15.4 %) compared with the skiers (4.5 %). Types III and IV spines were evenly distributed between both groups, and moreover, no differences were reported between the genders of both groups. The significant difference in type 1 spinal curvatures that were mentioned by Roussouly et al. [26] might suggest an association with the biomechanics and the muscular development required for function in sport [28–30]. Therefore, it could be hypothesised that a lack of abdominal, lumbar, pelvic and hip musculature tone within the control group might be a reason for their association with a ‘flat back’ type II spine.

Muyor et al. [42] showed no increment in the sagittal curvatures of adolescent tennis players. This was similar to the present study, where radiology values for the TK of the skiers (35.2°) and controls (37.4°) and LL of the skiers (−58.4°) and controls (−60.9°) were both similar. In the present study, radiological parameters were used to compare the spino-pelvic sagittal alignment; however, Uetake et al. [28], Alricsson and Werner [29], Rajabi et al. [30] and Muyor et al. [42] all used non-radiological methods. Agnvall et al. [31] and Barrett et al. [32] questioned the validity of non-radiological methods and demonstrated poor levels of agreement and a lack of sensitivity for correlation with radiological methods.

There are some limitations to the present study. Sagittal plane measurements were recorded in the erect standing position. However, this does not reflect the multi-directional patterns of skiing. In sport, repetitive fast trunk flexion and extension movements occur in the sagittal, frontal and transverse planes around the long axis of the spine [42]. Although a significant difference was shown for the classifications of type I spinal curvatures according to Roussouly et al. [26], this appeared different to analysing the spino-pelvic parameters between groups. Roussouly’s definition and classification of spinal types relates to analysing the entire spine. It may be possible that evaluation of spinal types according to Roussouly et al. [26] may be more sensitive and therefore show a difference in values compared to the evaluation of the spino-pelvic parameters. Other reasons may be related to the present study having such a small control group (*n* = 27) compared to the skiers (*n* = 75); perhaps, selection of a larger control group may have shown a greater difference in the values of the spino-pelvic parameters.

Spino-pelvic sagittal malalignment may develop during pubertal growth to accommodate postural and physiological changes that alter the spinal morphology [7, 11]. Osseous growth of the sacrum has been shown to occur up to and beyond 20 years of age [2, 43, 44]. Therefore, with the present study, by selecting a sample with the mean age of 17.7 years, spino-pelvic alignment differences might have been shown between groups due to postural and physiological changes associated with growth. The intentions of the present study were to have age-matched groups; however, a difference in age was shown between the skiers (18.3) and controls (16.4) and moreover was statistically significant in spite of both groups attending the same first year at Åre High School. Unfortunately, some skiers may have previously lived or studied abroad due to training and competition commitments and would have chosen to attend Åre High School because of the association with the Ski Academy. However, age differences and growth-related spurts amongst the skiers may have affected the spino-pelvic values and the outcome of the present study.

Other limitations include accuracy and interpretation of the radiological measurements. In the present study, spinal curvatures were calculated from measurements taken from the endplates of the vertebral bodies [4, 45–48], whilst the pelvic angles were calculated from measurements taken from the pelvic parameters [26]. Spinal posture can be affected by lower limb alignment [1, 49–52]; therefore, in the present study, errors may have occurred if the participants were not standing evenly in the same position, fatigued from prolonged periods of standing [53] or postural variance from biomechanical lower limb asymmetries [54]. In the present study, the inclusion criteria selected only a healthy population; however, this may have limited the ability to distinguish a greater difference between both groups.

The present study was able to show that type I spinal curvatures according to Roussouly et al. [26] was more common in young elite skiers compared with controls using radiological parameters. Therefore, the present study supports the hypothesis that the spino-pelvic sagittal alignment of young elite skiers is different to that of a healthy non-athletic population.

## Conclusion

The conclusions of the present study are that elite young skiers are shown to have a more prevalent type I spine and a different spino-pelvic sagittal alignment compared to a healthy non-sporting population of a similar age.

## Abbreviations

LL: lumbar lordosis
PI: pelvic incidence
PT: pelvic tilt
SVA: sagittal vertical axis
TK: thoracic kyphosis

## References

[1] Berthonnaud E, Dimner J, Roussouly P, Labelle H (2005). Analysis of the sagittal balance of the spine and pelvis using shape and orientation parameters. J Spine Disord.

[2] Descamps H, Commare M, Marty C, Hecquet J, Duval-Beaupere G (1999). Modifications des angles pelviens, don’t l’Incidence pelvinne, au cours de la croissance humaine. Biom Hum et Anthropol.

[3] Duval-Beaupere G, Schmidt C, Cosson P (1992). A barycentre-metric study of sagittal shape and pelvis: the conditions required for an economic standing position. Ann Biomed Eng.

[4] Roussouly P, Nnadi C (2010). Sagittal plane deformity: an overview of interpretation and management. Eur Spine J.

[5] Roussouly P, Pinheiro-Franco J (2011). Biomechanical analysis of the spino-pelvic organization and adaptation in pathology. Eur Spine J.

[6] Chanplakorn P, Wongsak S, Woratanarat P, Wajanavisit W, Loahacharoensombat W (2010). Lumbopelvic alignment on standing lateral radiograph of adult volunteers and the classification in the sagittal alignment of lumbar spine. Eur Spine J.

[7] Mac-Thiong J, Roussouly P, Bertonnaud E, Guigui P (2010). Sagittal parameters of global balance. Normative values from a prospective cohort of seven hundred and nine white asymptomatic adults. Spine.

[8] Schwab F, Lafage V, Patel A, Farcy JP (2009). Sagittal plane considerations and the pelvis in the adult patient. Spine.

[9] Mac-Thiong J, Labelle H, Bertonnaud E, Betz R, Roussouly P (2007). Sagittal spinopelvic balance in normal children and adolsecents. Eur Spine J.

[10] Boulay C, Tardieu C, Hecquet J, Mouilleseaux B, Marty C, Prat-Padal D, Legaye J (2006). Duval-Beaupère, Pélissier J. Sagittal alignment of spine and pelvis regulated by pelvic incidence: standard values and prediction of lordosis. Eur Spine J.

[11] Cil A, Yazici M, Uzumcugil A, Kandemir U, Alanay A, Alanay Y, Acaroglu R, Surat A (2005). The evolution of sagittal segmental alignment of the spine during childhood. Spine.

[12] Hammerberg E, Wood K (2003). Sagittal profile of the elderly. J Spinal Disord Tech.

[13] Jackson RP, Phipps T, Hales C, Surber J (2003). Pelvic lordosis and alignment in spondylolisthesis. Spine.

[14] Vendatam R, Lenke LG, Keeney JA, Bridwell KH (1998). Comparison of standing sagittal spinal alignment in asymptomatic adolescents and adults. Spine.

[15] Vialle R, Levassor N, Rillardon L, Templier A, Skalli W, Guigui P (2005). Radiographic analysis of the sagittal alignment and balance of the spine in asymptomatic subjects. J Bone Joint Surg Am.

[16] Kuntz C, Shaffrey CI, Ondra SL, Durrani AA, Mummaneni PV, Levin LS, Pettigrew DB (2008). Spinal deformity: a new classification derived from neutral upright spinal alignment measurements in asymptomatic juvenile, adolescent, adult, and geriatric individuals. Neurosurgery.

[17] Kuntz C, Levin L, ONdra S, Shaffrey C, Morgan C (2007). Neutral upright sagittal spinal alignment from the occiput to the pelvis in asymptomatic adults: a review and resynthesis of the literature. J of Neurosurgery.

[18] Boesker E, Moe J, Winter R, Koop S (2000). A determination of the normal thoracic kyphosis: a roentgengraphic study of 121 normal children. J Pediatr Orthop.

[19] Bridwell K, Bernhardt M (1989). Segmental analysis of the sagittal plane alignment of the normal thoracic and lumbar spines and thoracolumbar junction. Spine.

[20] Hardacker J, Shuford R, Capicotto R, Pryor P (1997). Radiographic standing cervical segmental alignment in adult volunteers without neck problems. Spine.

[21] Roussouly P, Gollogly S, Bertonnaud E, Dimnet J (2005). Classification of the normal variation in the sagittal alignment of the human lumbar spine and pelvis in the standing position. Spine.

[22] Jackson R, Kanemura T, Kawakami N, Hales C (2000). Lumbopelvic lordosis and pelvic imbalance on repeated standing lateral radiographs of adult volunteers and untreated patients with constant low back pain. Spine.

[23] Barrey C, Jund J, Noseda O, Roussouly P (2007). Sagittal balance of the pelvis-spine complex and lumbar degenerative diseases. A comparative study about 85 cases. Eur Spine J.

[24] Endo K, Suzuki H, Tanaka H, Kang Y, Yamamoto K (2010). Sagittal spinal alignment in patients with lumbar disc herniation. Eur Spine J.

[25] Marty C, Boisaubert B, Descamps H, Montigny JP, Hecquet J, Legaye J, Duval-Beaupere G (2002). The sagittal anatomy of the sacrum among young adults, infants, and spondylolisthesis patients. Eur Spine J.

[26] Roussouly P, Berthonnaud E, Dimnet J (2003). Geometrical and mechanical analysis of lumbar lordosis in an asymptomatic population: proposed classification. Rev Chir Orthop Reparatrice Appar Mot.

[27] Van Royen BJ, Toussaint HM, Kingma I, Bot SD, Caspers M, Harlaar J, Wuisman PI (1998). Accuracy of the sagittal vertical axis in a standing lateral radiograph as a measurement of balance in spinal deformities. Eur Spine J.

[28] Uetake T, Ohtsuki F, Tanaka H, Shindo M (1998). The vertebral curvature of sportsmen. J Sports Sci.

[29] Alricsson M, Werner S (2006). Young elite cross-country skiers and low back pain. A 5-year study. Phys Ther Sport.

[30] Rajabi R, Doherty P, Goodarzi M, Hemayattalab R (2007). Comparison of thoracic kyphosis in two groups of elite Greco- Roman and free style wrestlers and a group of non-athletic subjects. Br J Sports Med.

[31] Agnvall C, Todd C, Kovac P, Swärd A, Baranto A, Swärd L, Karlsson J. Validation of spinal sagittal alignment with plain radiographs and the Debrunner Kyphometer. Medical Research Archives. 2015;2(1). doi:http://dx.doi.org/10.18103/mra.v2i1.319.

[32] Barrett E, McCreesh K, Lewis J (2014). Reliability and validity of nonradiographic methods of thoracic kyphosis measurement: a systematic review. Man Ther.

[33] Brennan P, McDonnell S, O’Leary D (2004). Increasing film-focus distance (FFD) reduces radiation dose for x-ray examinations. Radiat Prot Dosimetry.

[34] Legaye J, Duval-Beaupere G, Hecquet J, Marty C (1998). Pelvic incidence: a fundamental pelvic parameter for three-dimensional regulation of spinal sagittal curves. Eur Spine J.

[35] Boulay C, Tardieu C, Hecquet J (2005). Be’ n- aim C, Mitulescu A, et al. Anatomical reliability of two fundamental radiological and clinical pelvic parameters: incidence and thickness. Accepted Eur J Orthop Surg Traumatol.

[36] Guigui P, Levassor N, Rillardon L, Wodecki P, Cardinne L (2003). Physiological value of pelvic and spinal parameters of sagittal balance: analysis of 250 healthy volunteers. Rev Chir Orthop Reparatrice Appar Mot.

[37] Itoi E (1991). Roentgenographic analysis of posture in spinal osteoporotics. Spine.

[38] Vaz G, Roussouly P, Berthonnaud E, Dimnet J (2002). Sagittal morphology and equilibrium of pelvis and spine. Eur Spine J.

[39] Voutsinas S, MacEwen G (1986). Sagittal profiles of the spine. Clin Orthop.

[40] Willner S (1981). Spinal pantograph: a non-invasive technique for describing kyphosis and lordosis in the thoraco-lumbar spine. Acta Orthop Scand.

[41] Mac-Thiong J, Labelle H, Roussouly P (2011). Pediatric sagittal alignment. Eur Spine J.

[42] Muyor J, Sánchez-Sánchez E, Sanz-Rivas D, Lòpez-Miñarro P (2015). Sagittal spinal morphology in highly trained adolescent tennis players. J of Sports Science and Medicine.

[43] Descamps H, Commare MC, Marty C, Duval-Beaupere G (1996). Le parame’ tre Incidence chez le petit enfant. Rachis.

[44] Mangione P, Gomez D, Senegas J (1997). Study of the course of the incidence angle during growth. Eur Spine J.

[45] Mannion AF, Knecht K, Balaban G, Dvorak J, Grob D (2004). A new skin-surface device for measuring the curvature and global and segmental ranges of motion of the spine: reliability of measurements and comparison with data reviewed from the literature. Eur Spine J.

[46] Cobb JR (1948). Outline for the study of scoliosis. Instr Course Lect.

[47] Singer KP, Edmondston SJ, Day RE, Breidahl WH (1994). Computer-assisted curvature assessment and Cobb angle determination of the thoracic kyphosis. Spine.

[48] Harrison DE, Cailliet R, Harrison DD, Janik TJ, Holland B (2002). Reliability of Centroid, Cobb and Harrison posterior tangent methods: which to choose for analysis of thoracic kyphosis. Spine.

[49] During J, Goudfrooij H, Keesen W, Beeker TW, Crowe A (1985). Toward standards for posture: postural characteristics of the lower back system in normal and pathologic conditions. Spine.

[50] Farcy JP, Schwab FJ (1997). Management of flatback and related kyphotic decompensation syndromes. Spine.

[51] Horton WC, Brown CW, Bridwell KH (2005). The effect of arm position on sagittal plane alignment. Spine.

[52] Kim KT, Suk KS, Cho YJ, Hong GP, Park BJ (2002). Clinical outcome results of pedicle subtraction osteotomy in ankylosing spondylitis with kyphotic deformity. Spine.

[53] Hinman MR (2004). Interrater reliability of flexicurve postural measures among novice users. J Back Musculoskelet Rehabil.

[54] D’Osualdo F, Schierano S, Iannis M (1997). Validation of clinical measurement of kyphosis with a simple instrument, the arometer. Spine.

