# Erratum to: Comparison of radiological spino-pelvic sagittal parameters in skiers and non-athletes

**DOI:** 10.1186/s13018-016-0485-8

**Published:** 2016-11-22

**Authors:** Carl Todd, Peter Kovac, Anna Swärd, Cecilia Agnvall, Leif Swärd, Jon Karlsson, Adad Baranto

**Affiliations:** 1 Department of Orthopaedics, University of Gothenburg and Sahlgrenska University Hospital, Gothenburg, Sweden; 2 The Carl Todd Clinic, 5 Pickwick Park, Park Lane, Corsham, SN13 0HN UK; 3 Department of Radiology, Institute of Clinical Sciences at Sahlgrenska Academy, University of Gothenburg and Sahlgrenska University Hospital, Gothenburg, Sweden; 4 Sportsmedicine Åre and Åre Ski High School, Gothenburg, Sweden

## Erratum

After publication of the original article [1], it was brought to our attention that the measurement of the Sagittal Vertebral Axis (SVA) noted throughout the text as “cm” should have read “mm”. We apologise for any confusion this may have caused.
